# Supplementary material for: The role of polygenic susceptibility to obesity among carriers of pathogenic mutations in MC4R in the UK Biobank population
Source: PLoS Med. 2020 Jul 21;17(7):e1003196. doi: 10.1371/journal.pmed.1003196 (PMC7373259; doi:10.1371/journal.pmed.1003196)
Supplement: S1 Text — (PDF) [file pmed.1003196.s001.pdf]

The role of polygenic susceptibility to obesity among carriers of pathogenic mutations in MC4R in the UK Biobank population

Nathalie Chami[1,2], Michael Preuss[1,2], Ryan W. Walker[3], Arden Moscati[1], Ruth J.F. Loos[1,2,3,*]

1. The Charles Bronfman Institute for Personalized Medicine, Icahn School of Medicine at Mount Sinai, New York, NY, USA

2. The Mindich Child Health and Development Institute, Icahn School of Medicine at Mount Sinai, New York, NY, USA

3. Department of Environmental Medicine and Public Health, Icahn School of Medicine at Mount Sinai, New York, NY, USA

* Ruth.loos@mssm.edu

1. CONTENTS

2. Phenotype data....2
3. Identifying Individuals of European Ancestry....3
4. Penetrance and odds ratio to select high-impact mutations 3
5. Polygenic risk score for BMI....3
6. Quality control of genotyped data of the 69 MC4R mutations....4
7. Sensitivity Analyses....5
8. References....6

2. PHENOTYPE DATA

All phenotype data used for our analyses was collected at the participants' baseline visit. Body mass index (BMI), calculated as weight (kg) divided by height (m^2) squared, was used to identify individuals who are underweight (BMI<18.5 kg/m^2) normal weight (18.5 kg/m^2 ≤ BMI < 25 kg/m^2), overweight (25 kg/m^2 ≤ BMI < 30 kg/m^2), or obese (BMI ≥ 30 kg/m^2). Height was measured standing without shoes, using a Seca 202 device. Weight was measured, after removing shoes and heavy outer clothing, using the Tanita BC-418 MA body composition analyzer.
Waist-to-hip ratio (WHR) was calculated by dividing waist over hip circumference, which were measured using a using a Wessex non-stretchable sprung tape. Body composition (fat mass and fat free mass) and basal metabolic rate was assessed by bio-impedance, using the Tanita BC-418MA body composition analyzer. Body fat percentage (BF%) was calculated as fat mass (kg) divided by weight (kg) times 100. We calculated the fat free mass index (FFMI) as fat free mass (kg) divided by height (m^2) squared, to assess relative leanness [1].

Participants' "comparative body size at age 10" and "comparative height size at age 10" was obtained through questionnaire. Participant's answer the question "When you were 10 years old, compared to average would you describe yourself as:" thinner, about average, or plumper for body size and shorter, about average, or taller for height. Birth weight (kg) in men and women and age at menarche (y) for women were obtained through self-report.

Daily physical activity was assessed using the International Physical Activity Questionnaire (IPAQ) [2]. Participants completed the short form of the questionnaire, which allows inferring a continuous MET (metabolic equivalent task) score. METs are multiples of resting metabolic rate and MET-minutes are computed by multiplying the MET score of an activity by the minutes performed. We used the summed MET-minute/week for all activities (including walking, moderate and vigorous activity), as an overall estimate of daily physical activity. Besides a continuous score, IPAQ allows categorizing individuals in three groups of physical activity levels. The grouped labeled as "high" includes individuals are either a) active at vigorous-intensity on at least 3 days, achieving ≥1,500 MET-minutes/week, or b) walk, or do moderate-intensity or vigorous-intensity activities, achieving ≥3,000 MET-minutes/week. The grouped labeled as "moderate" includes individuals who participate in a) vigorous-intensity activities ≥3 days a week for ≥20 minutes per day, or b) moderate-intensity activities and/or walking for ≥5 days for ≥30 minutes per day, or c) any combination of walking, moderate-intensity or vigorous intensity activities achieving a ≥600 MET-minutes/week.

The Townsend Deprivation Index (TDI), a proxy of socioeconomic status, was calculated immediately prior to participation, based on the participant's postcode. It is a composite measure of deprivation based on the level of social deprivation in which the participant lives; a negative value represents high socio-economic position.

3. IDENTIFYING INDIVIDUALS OF EUROPEAN ANCESTRY

We identified individuals of European ancestry based on their genetic information, using k-means clustering. First, we calculated principal components and their loadings for all 488,377 genotyped UK Biobank participants based on the intersection of ~121,000 QC'ed UK Biobank variants and 1000G Phase 3 v5 reference panel. Reference ancestries comprised a total of 2,504 individuals: 503 were European (EUR), 347 American Admixed (AMR) 661 African (AFR), 504 East Asian (EAS) and 489 South Asian (SAS) samples. We projected the 1000G reference panel dataset based on the calculated PCA loadings from the UK Biobank. We then used k-means clustering with a pre-specified amount of 4 clusters to the UK Biobank PCA and the projected 1000G reference panel dataset. Individuals from UK Biobank which were clustered within the most EUR individual cluster from the 1000G reference panel where assigned as individuals with European ancestry resulting in 453,812 UK Biobank samples identified as individuals of European ancestry.

4. PENETRANCE AND ODDS RATIO TO SELECT HIGH-IMPACT MUTATIONS

Penetrance and OR are correlated (**Fig 2**), yet they are not redundant, but rather complimentary. Specifically, penetrance is calculated as [(# carriers with obesity/total # of carriers) x 100], whereas the OR is calculated as the odds of mutation carriers to have obesity compared to them being of normal weight and the odds of non-carriers to have obesity compared to them being of normal weight. Thus, in the penetrance calculation, only carriers are considered, and the denominator includes carriers with obesity, overweight and normal weight. The OR calculation, includes carriers and non-carriers, and those with obesity are compared to those of normal weight, whereas individuals with overweight are not considered. OR and penetrance will differ for mutations that have e.g. a relatively high percentage of obese (high penetrance), but when the ratio between obesity/normal weight is similar to that of non-carriers (i.e ~0.75), the OR nears 1 (e.g. rs13447335). Vice versa, the OR can be high, because the obesity/normal weight ratio in carriers is higher than in non-carriers, but the percentage of individuals with obesity among carriers (penetrance) may be low, because there's a substantial number of overweight (and normal weight) (e.g. rs762251697).

Since the prevalence of obesity in the UKBB is 25%, we chose a cutoff of 30% for penetrance to select mutations that are more penetrant than what is expected based on population prevalence. Furthermore, as the ratio of obesity over normal weight among non-carriers in the UK Biobank is 0.74 (i.e. more individuals of normal weight than individuals with obesity), mutations that increase the odds of obesity by twofold or more (compared to normal weight) were considered to have a moderate-to-high risk.

As can be seen in **Fig 2**, each parameter on its own (i.e. penetrance >30%, or OR >2) would identify more mutations, but by combining them, we are more stringent and identify a subset of mutations that we consider to have higher impact than when only one parameter would be used.

5. POLYGENIC RISK SCORE FOR BMI

We calculated a PRS for BMI (PRS_{BMI}) using the software PRSice [3] and summary statistics from the most recent GWAS meta-analyses for BMI [4] that does not include data from the UK Biobank. Samples with missingness rate > 90% were removed. In addition, we removed variants with a MAF ≤5%, a call rate <95% and imputation quality info-score of <0.40 and subsequently retained variants for which association with BMI reached P<0.5 and LD was r^2 <0.8. Our PRS_{BMI} included

351,597 variants and explained 7% of the phenotypic variation in BMI. Each standard deviation increase in PRS_{BMI}, was associated with a 1.29 kg/m^2 increase in BMI (equivalent to ~3.7 kg/SD for a 1.7m-tall person, $P < 2x10^{-16}$). The correlation between our PRS_{BMI} and BMI was 0.26, consistent with what was recently reported (correlation = 0.29) [5].

6. QUALITY CONTROL OF GENOTYPED DATA OF THE 69 MC4R MUTATIONS

6.1. Inspection of cluster plots

We generated cluster plots using Evoker [6,7] for all 69 variants. Specifically, there are 107 batches per variant, such that we visualized 7,383 cluster plots (i.e. 69 variants x 107 batches) for manual inspection by three investigators. Cluster plots with carriers can be viewed here: http://bit.ly/34cZc0K.

As described in [8] and [9], we inspected the quality of the separation between heterozygous carriers and homozygous non-carriers and rated the clustering for each variant as "poor", "intermediate", or "good". A variant was deemed of "poor" quality, if the majority of clusters had an unclear or poor separation between the two clusters, of "intermediate" quality, if a substantial number of plots showed unclear separation, and of "good" quality, if all or the vast majority of the clusters had a clear separation [9]. We then summarized the overall quality, based on the assessment of each of the three investigators (S2 **Table,** columns T-W). As such, we identified 10 variants of low quality ("poor" or "intermediate") (S2 **Table**, columns Y), that were therefore deemed to be of unacceptable quality and were removed from downstream analyses. All the 11 *MC4R* mutations with high penetrance and effect were of high quality.

6.2. Comparison of genotyped and sequenced data

We took advantage of the UK Biobank whole exome sequencing data, available for a subset of 50,000 individuals [10]. We examined the concordance between the genotyped and sequenced data in the subset of ~46,000 individuals of European ancestry for whom both data sources were available (S2 **Table**, columns J-O).

Among the 46,000 individuals of European ancestry that were sequenced,

- 25 of the 69 genotyped variants had no carriers in the genotyped data or in the sequenced data. This would suggest that sequenced and genotyped data were concordant (i.e. 100% concordance, no false positive observations).
- 44 of the 69 genotyped variants had at least one carrier in the genotyped data, which we expected to observe in the sequenced data. Of these 44 variants,
    - o carriers were indeed observed in the sequenced dataset for 21 variants, with variable concordance (**see below**);
    - o for the 23 other variants, we did not observe carriers in the sequenced data, suggesting that all genotyped carriers could be false positive observations.

For each mutation, we calculated the **false positive proportion** (FPP; i.e. number of carriers observed in genotyped data minus number of carriers observed in sequencing data, divided by total number of carriers observed genotyped data), and the **false negative proportion** (FNP; number of carriers observed in sequenced data minus number of carriers observed in genotyped data, divided by total number of genotyped carriers) (S2 **Table**).

Of the 21 variants for which carriers were observed in both genotype and sequencing datasets (S2 **Table**, columns P-Q),

- for 13 variants, there was 100% concordance between the two platforms; i.e. the FPP = 0%;
- for 4 variants, the FPP was between >0% and ≤25% and deemed acceptable;
- for 4 variants, the FPP >25% and deemed unacceptable.

For the 48 variants (see above 25 + 23 variants) for which no carriers were observed in the

sequencing data,

- the 26 variants with no carriers in either genotyping and sequencing data are considered 100% concordant or FPP = 0%, and thus of acceptable quality,
- whereas the 22 variants with genotyped carriers that were not observed in the sequencing data would be considered all false positives (FPP = 100%), and deemed of unacceptable quality.

Across all 69 genotyped variants, the false negative proportion (FNP) was >25% for 3 variants (i.e. of unacceptable quality) and ≤25% for all others (S2 **Table**, columns R-S).

Thus, based on the comparison between genotyping and sequencing data in the subset of ~46,000 individuals, we identified at least 4 variants with an unacceptable FPP (>25%), another 22 variants for which the FPP was calculated to be 100%, and 3 variants (one that overlapped with the 4 variants above) with an unacceptable FNP (>25%). Thus, the quality of the genotyped data for 28 variants was deemed to be low, based on the comparison in the 46,000 participants.

It is important to acknowledge that using FPP and FNP to assess concordance between the genotyped and sequenced data has its limitations. Specifically, the FPP and FNP have little or no resolution and may result in exaggerated values when carriers are rare. E.g. if there is 0 or only 1 carrier, the FPP will be either 0 or 100%; no values between 0 and 100% are possible. Furthermore (and related to the low resolution), FPPs calculated for rare variants in a relatively small subset of ~46,000 individuals are not stable and may not scale up to the full population of ~450,000 individuals.

6.3. Summary

In sum, we identified 10 variants of which the cluster plots for the full cohort (N~450,000) were considered to be of "unacceptable" quality, and 28 variants for which there was unacceptable discordance between genotyped and sequenced data in a subset of ~46,000 individuals.

We have removed the 10 variants with poor cluster plots from analysis, leaving 59 variants in our study.

For the 28 variants with genotyping discordance, we also considered their cluster plots, because of the limitations of the FPP/FNP metric and because the sequenced subset consists of only ~10% of the full population analyzed. For 20 of these 28 variants, the cluster plots across the ~450,000 individuals are of good quality. Therefore, we flagged these 20 variants, 3 of which are among the high impact mutations (S2 **Table**, column X), and assessed their impact in a sensitivity analysis in which we remove them.

7. SENSITIVITY ANALYSES

Based on inspection of the cluster plots, all 11 variants were deemed of "good quality", but two variants (rs1367004987, Affx-89021050) have high FPPs (>25%) and one variant (rs775382722) has a high FNP (>25%), based on the comparison between the genotyped and sequenced data in the 46K subset. Because this misclassification may have influenced our findings, we performed a sensitivity analysis in which excluded all three variants rs1367004987, Affx-89021050, and rs775382722.

After excluding rs1367004987, Affx-89021050 and rs775382722, our sensitivity analysis – with 8 high-impact variants left – shows that the mean (SD) PRS values for carriers and non-carriers in individuals of normal weight and with obesity continue to be robust (**S1 Fig, S2 Fig, S3 Table**). Because the sample size of carriers is smaller and SE a little larger, P-values are a little higher (P_{sens} = 1 x 10^{-4} vs P_{orig} = 1.7 x 10^{-6}). Differences for other characteristics remained very similar as well (**S3 Table**). Furthermore, the additive effects of the 8 high-impact mutations and PRS on BMI are nearly the same (**S3 Fig**).

8. REFERENCES

1. VanItallie TB, Yang MU, Heymsfield SB, Funk RC, Boileau RA. Height-normalized indices of the body's fat-free mass and fat mass: potentially useful indicators of nutritional status. Am J Clin Nutr. 1990; 52:953-9.
2. Guidelines for Data Processing and Analysis of the International Physical Activity Questionnaire (IPAQ) [Available from: http://biobank.ctsu.ox.ac.uk/crystal/docs/ipaq_analysis.pdf.
3. Euesden J, Lewis CM, O'Reilly PF. PRSice: Polygenic Risk Score software. Bioinformatics. 2015; 31:1466-8.
4. Locke AE, Kahali B, Berndt SI, Justice AE, Pers TH, Day FR, et al. Genetic studies of body mass index yield new insights for obesity biology. Nature. 2015; 518:197-206.
5. Khera AV, Chaffin M, Wade KH, Zahid S, Brancale J, Xia R, et al. Polygenic Prediction of Weight and Obesity Trajectories from Birth to Adulthood. Cell. 2019; 177:587-96 e9.
6. Morris JA, Randall JC, Maller JB, Barrett JC. Evoker: a visualization tool for genotype intensity data. Bioinformatics. 2010; 26:1786-7.
7. Bycroft C, Freeman C, Petkova D, Band G, Elliott LT, Sharp K, et al. The UK Biobank resource with deep phenotyping and genomic data. Nature. 2018; 562:203-9.
8. Wright CF, West B, Tuke M, Jones SE, Patel K, Laver TW, et al. Assessing the Pathogenicity, Penetrance, and Expressivity of Putative Disease-Causing Variants in a Population Setting. Am J Hum Genet. 2019; 104:275-86.
9. Lotta LA, Mokrosiński J, Mendes de Oliveira E, Li C, Sharp SJ, Luan J, et al. Human gain-of-function MC4R variants show signaling bias and protect against obesity. Cell. 2019.
10. Hout CVV. Whole exome sequencing and characterization of coding variation in 49,960 individuals in the UK Biobank. biorxiv. 2019.
