# Supplementary figures and images for: The role of polygenic susceptibility to obesity among carriers of pathogenic mutations in MC4R in the UK Biobank population
Source: PLoS Med. 2020 Jul 21;17(7):e1003196. doi: 10.1371/journal.pmed.1003196 (PMC7373259; doi:10.1371/journal.pmed.1003196)

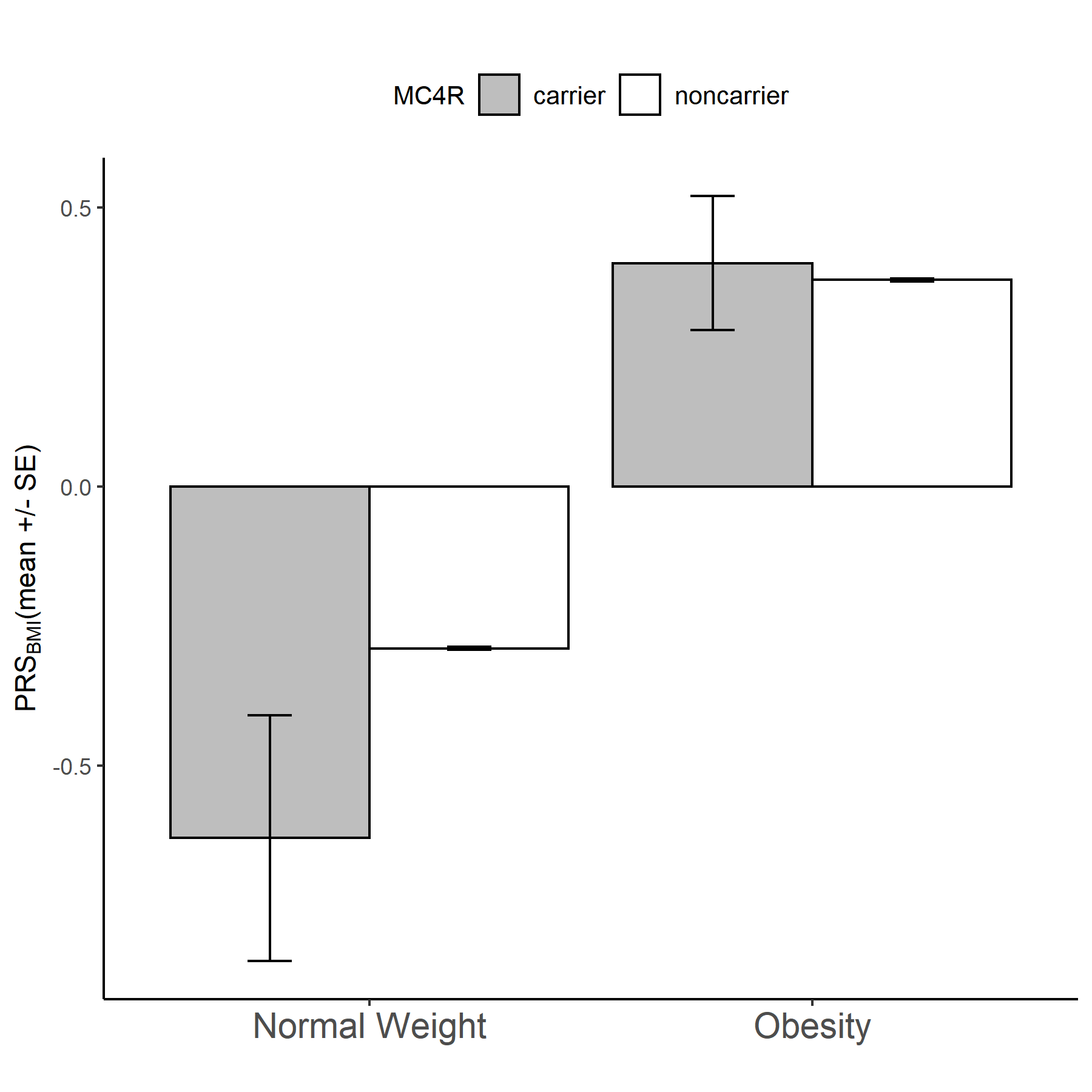

Supplement: S1 Fig — PRSBMI, polygenic risk score for BMI. (TIF) [file pmed.1003196.s002.tif]

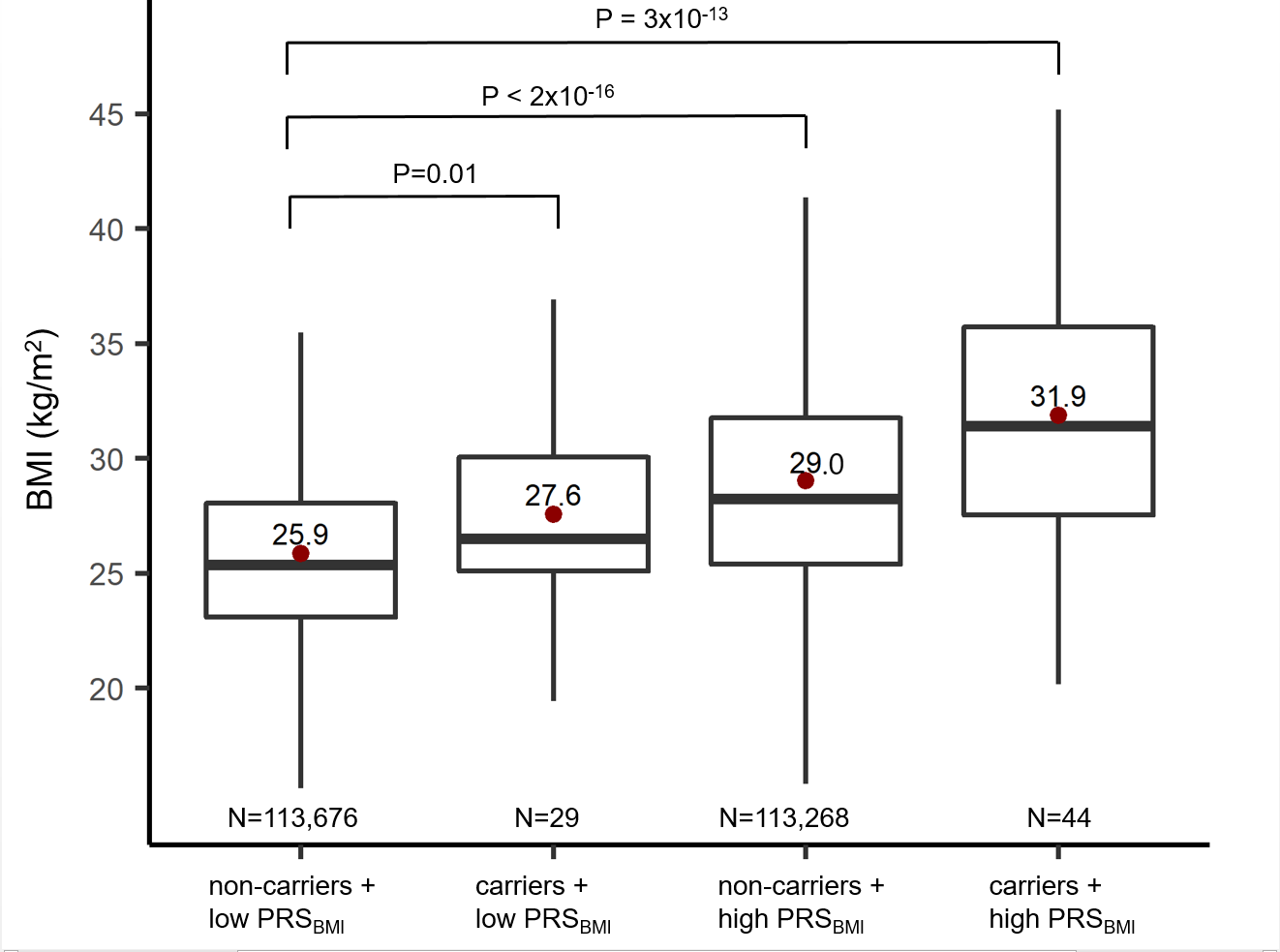

Supplement: S2 Fig — BMI, body mass index; PRSBMI, polygenic risk score for BMI. (TIF) [file pmed.1003196.s003.tif]

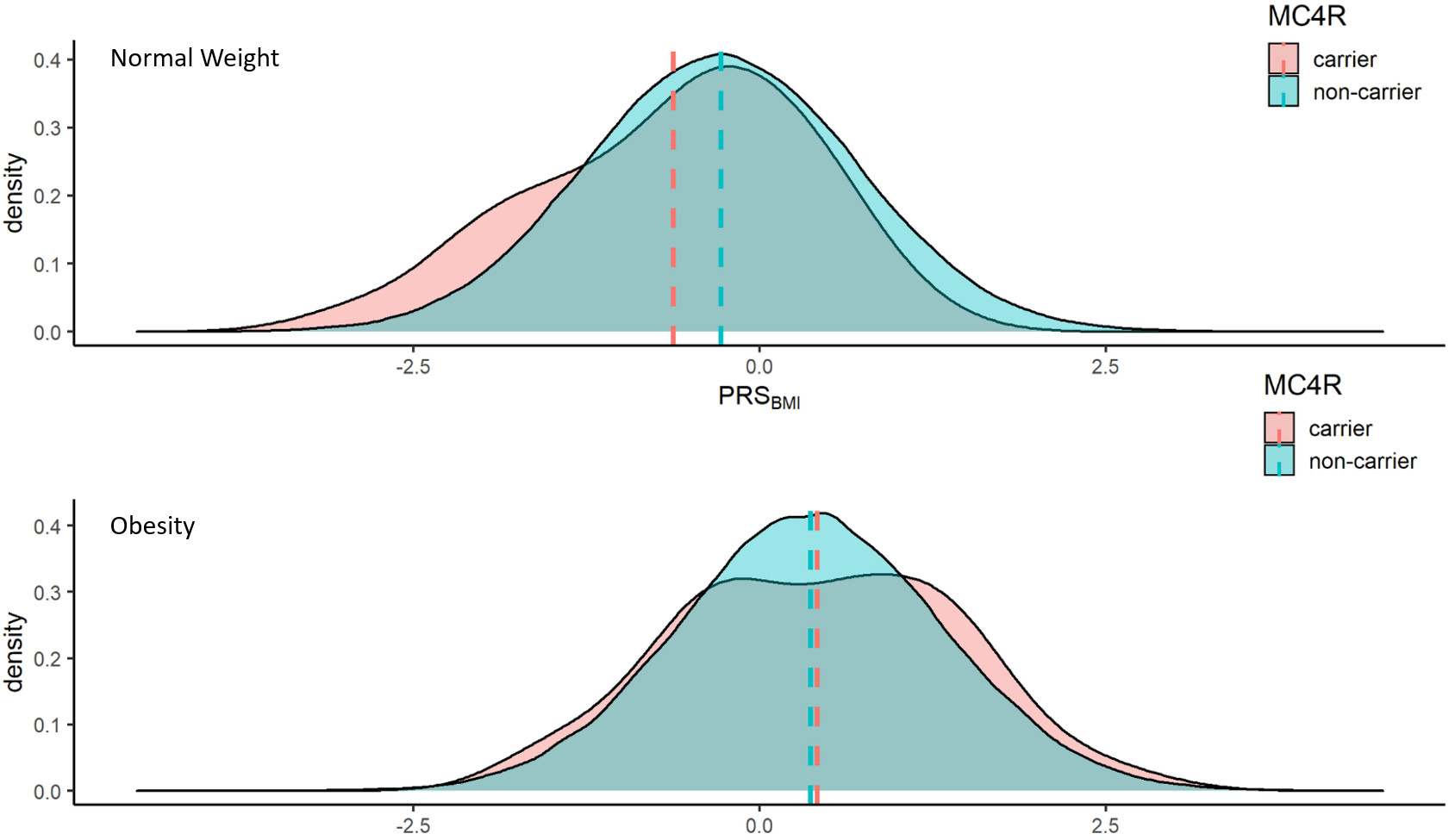

Supplement: S3 Fig — MC4R, melanocortin 4 receptor gene; PRSBMI, polygenic risk score for BMI. (TIF) [file pmed.1003196.s004.tif]
