# Supplementary material for: The role of polygenic susceptibility to obesity among carriers of pathogenic mutations in MC4R in the UK Biobank population
Source: PLoS Med. 2020 Jul 21;17(7):e1003196. doi: 10.1371/journal.pmed.1003196 (PMC7373259; doi:10.1371/journal.pmed.1003196)
Supplement: Attachment — Submitted filename: PMEDICINE-D-20-00082R1 - RESPONSE TO EDITOR AND REVIEWERS.pdf [file pmed.1003196.s013.pdf]

RESPONSE TO EDITOR AND REVIEWERS

Requests from the editor:

- ***Title***

Comment: *Please revise your title according to PLOS Medicine's style. Your title must be nondeclarative and not a question. It should begin with main concept if possible. "Effect of" should be used only if causality can be inferred, i.e., for an RCT. Please place the study design ("A randomized controlled trial," "A retrospective study," "A modelling study," etc.) in the subtitle (ie, after a colon).*

Response: We have changed the title accordingly to "The role of polygenic susceptibility to obesity among carriers of pathogenic mutations in *MC4R*" (**P1**).

- ***Abstract***

Comment: *Background - is it monogenic forms of obesity? Please specify this point if so*

Response: Yes, it is monogenic forms of obesity. We have updated the abstract accordingly (**P2 L3**).

Comment: *Background - please clearly highlight the aim of your study*

Response: We have updated the abstract accordingly (**P2 L4-7**).

Comment: *Format - Please structure your abstract using the PLOS Medicine headings (Background, Methods and Findings, Conclusions).*

Response: We have updated the abstract accordingly.

Comment: *Please combine the Methods and Findings sections into one section, "Methods and findings".*

Response: We have combined methods and results in a reorganized "Methods and Findings" section.

Comment: *Please include the study design, population and setting, number of participants, years during which the study took place, length of follow up, and main outcome measures*

Response: We have updated the abstract accordingly.

Comment: *Please provide 95% confidence intervals along with p values*

Response: Consistent with the tables and text, we have added standard errors to the mean values and P-values

Comment: *Please clarify this sentence "Normal weight carriers more often reported that, already at age 10y, they were thinner/average (72%) compared to obese carriers (48%) (P=0.02)". From the language it appears as though the weight was self reported which should be clarified in the methods section of the abstract. Also it is not clear if the measure is BMI or body composition, Please revise as needed.*

Response: Body weight and height in adulthood were measured in a research setting using standardized methods. Participants were additionally asked about how their "body size" compared to peers at age 10yrs (thinner/average/plumper), which was self-reported. We have further clarified this sentence in the abstract.

❖ ***Abstract conclusions***

Comments:

- *Please address the study implications without overreaching what can be concluded from the data; the phrase "In this study, we observed ..." may be useful.*
- *Please interpret the study based on the results presented in the abstract, emphasizing what is new without overstating your conclusions.*
- *Please avoid vague statements such as "these results have major implications for policy/clinical care". Mention only specific implications substantiated by the results.*
- *Please avoid assertions of primacy ("We report for the first time....")*

Response: We have updated the abstract accordingly.

❖ ***Author summary***

Comments: *At this stage, we ask that you include a short, non-technical Author Summary of your research to make findings accessible to a wide audience that includes both scientists and non-scientists. The Author Summary should immediately follow the Abstract in your revised manuscript. This text is subject to editorial change and should be distinct from the scientific abstract. Please see our author guidelines for more information: https://urldefense.proofpoint.com/v2/url?u=https-3A__journals.plos.org_plosmedicine_s_revising-2Dyour-2Dmanuscript-23loc-2Dauthor-2Dsummary&d=DwIGaQ&c=shNJtf5dKgNcPZ6Yh64b-A&r=GjDfTbqtpuszLNb8BNrmfuY1BR0v0jOtKmdjadhZVas&m=Xz57CVACEKH9U4QZtW31CbDST6uPkwdLtGnFoLjPH9s&s=jrH5HWOfckbuVuF2EQDsKiKgftXDA_Kh-tfG6P7EjCc&e=*

Response: We have included an “Author Summary” (**P 4-5**)

❖ ***Introduction***

Comments*: References must be in Vancouver style and provided within square brackets please.*

Response: We have updated references and citations accordingly

Comment*: Paragraph 3 on page 4- I assume this is still MC4R mutations so it might be better to mention this in the first two sentences*

Response: We have added that the mutation is in *MC4R* (**P6 L19**).

Comment*: Please move methods to just after Introduction on page 5*

Response: We have moved the methods section to just after the Introduction (**P7-12**).

❖ ***Results***

Comment*: Please simplify this sentence: “For 10 (91%) of the 11 mutations, there was evidence that the mutation impaired MC4R function and/or led to reduced activity, based on functional characterization (Tables S3 and S4), which was significantly more often than for the remaining 48 mutations (P = 0.0006) for which we found evidence for only 17 (33%)”. You may consider splitting up the sentence as it is currently hard to follow.*

Response: We have split the sentence in two shorter and simplified sentences (**P13 L4-7**).

Comment*: Please report p values of up to two decimal places*

Response: We have made changes throughout the text accordingly.

***Comment**: Please provide "(no individuals carried more than one mutation)" as a separate sentence.*

Response: We have made changes to the text accordingly (**P13 L10**).

***Comment**: Page 7 please introduce FFMI on first view*

Response: As we have moved the "Methods" section before the "Results" section, fat free mas index (FFMI) is first introduced at **P8 L9**.

❖ ***Discussion***

***Comment:** Please rephrase "beating their genetic odds" on page 8. The same goes for "counteract the obesity-increasing effects of MC4R mutations".*

Response: We have removed the "beating their genetic odds" phrase and rephrased the following sentence (**P15 L21-22**).

***Comment:** Recommend revising instances "normal phenotype" to non-obese or similar, to avoid any stigmatising labels.*

Response: We agree and have changed the text (**P17 9-10**).

***Comment:** Please avoid assertions of primacy such as "we show for the first time" by adding "to our knowledge"*

Response: We have removed this "first time" assertion.

***Comment:** You mention sensitivity analyses here but not in the results? Please provide these as SI files as needed to support your findings*

Response: We reported the sensitivity analyses in the Methods section, which has been moved before the Results and Discussion sections, at **P10 L7**.

***Comment:** Limitations of the UK biobank cohort and your methodology (specifically self reported weight) more generally must be outlined in further detail.*

Response: Body weight and height were measured in a clinical/research setting using standardized procedures. We provide more detail on data collection in the **Supplemental Information** under the "Phenotype" section: "Weight was measured, after removing shoes and heavy outer clothing, using the Tanita BC-418 MA body composition analyzer". Body size at age 10y was obtained through questionnaire. We describe this phenotype in the same section of the **Supplemental Information**.
We acknowledge the limitations of the UK Biobank cohort in the next to last paragraph of the Discussion section (**P18-19**).

***Comment:** Page 22- please revise the last sentence containing multiple instances of (R,2013)*

Response: We have corrected this (**P12 L6**).

***Comment:** Please ensure that the study is reported according to a appropriate reporting guideline (GRIPS? Or STROBE), and include the completed checklist as Supporting Information. When completing the checklist, please use section and paragraph numbers, rather than page numbers.*

Response: We have reported the study according to **STROBE.**

Comment: *Please add the following statement, or similar, to the Methods: "This study is reported as per the xxxx guideline (S1 Checklist)."*

Response: We have added this sentence to the Methods section (**P12 L7**).

Comment: *Please report your study according to the relevant guideline, which can be found here: https://urldefense.proofpoint.com/v2/url?u=http-3A__www.equator-2Dnetwork.org_&d=DwIGaQ&c=shNJtf5dKgNcPZ6Yh64b-A&r=GjDfTbqtpuszLNb8BNrmfuY1BR0v0jOtKmdjadhZVas&m=Xz57CVACEKH9U4QZtW31CbDST6uPkwdLtGnFoLjPH9s&s=yfpTUYDZvbZc1W0QXdfSbnSGX4ig3iVHPj1e5gECBtc&e=*

Response: We have reported the study according to STROBE**.**

Additional note:

In reviewing our data, we noticed that with the OPCS-4-codes used, we had not excluded all 274 participants that had undergone bariatric surgery, one of which was a normal weight carrier. We subsequently performed all relevant analyses again. This did not materially change on our results or conclusions. We have updated all relevant text, tables and figures to reflect this change.

Reviewer 1:

Comment 1: *One aspect is that I am not sure the second aim of the study was fully answered on why some individuals who carry these mutations are able to remain of normal weight - largely the results simply report the observed differences between groups and does not investigate the underlying effect modification between environmental factors in each group. For instance, on page 7, the authors state that environmental/lifestyle (non-genetic factors were examined). They have provided some interesting results in on Table 1 comparing lifestyle factors stratifying by carriers and non-carriers. What I find interesting is that there is some definite effect modification by lifestyle factors here. For instance, physical activity (MET and IPAQ) are not different between obese and normal weight carriers but is significantly different in normal and obese non-carriers. Also other demographic attributes like sex and height look to have important differences in association between normal and obese in carriers vs non-carriers. Could the authors elaborate further on whether environmental factors looks like they might have a pretty strong modifying effect - potentially even more so than genetic susceptibility and whether there were any interactions that could be explore in the analysis between gene and environment.*

Response 1: We agree with the reviewer that the inspection of environmental factors and their contribution to lowering the risk of obesity is of great interest and so is the exploration of gene-environment interactions. Indeed, the second aim of our study was to explore whether there are other genetic or non-genetic/environmental factors that would impact the BMI of mutation carriers.

As suggested, we examined the contribution of non-genetic/environmental factors, including physical activity and socioeconomic status. Unfortunately, accurate dietary information was not available for sufficient participants (in particular carriers) for statistically powered analyses (**P8 L11-19 and Supplemental Information**). We describe these difference in the results section (**P14 L22-25**). Specifically, carriers of normal weight seem to have been exposed/have experienced a healthier environment than carriers who are obese. We elaborate on the contribution of factors, genetic and non-genetic, that may explain the difference between carriers of normal weight and those with obesity in the Discussion section (**P17 L12 – P18 L9**). However, a key limitation in assessing the contribution of non-genetic/environmental factors is that the majority of data was collected cross-sectionally, which does not allow determining whether the

healthier environment was indeed the cause of the lower body weight, or whether it was the consequence. We acknowledge this limitation in the Results (**P14 L24-25)** and Discussion (**P18 L19-22)** sections.

The reviewer correctly noted that for some phenotypes (e.g. physical activity, height, sex), differences between normal weight vs obese carriers are not significant, whereas they are significantly different in non-carriers. However, for this type of comparisons, it is important to also compare effect sizes, as the sample size for non-carriers is enormous, such that even the smallest difference will be highly significant. For example, for physical activity, the difference between carriers of normal weight vs those with obesity is ~0.43 SD in MET, reaching P=0.07 with 84 individuals. Among non-carriers this difference is ~0.32 SD in MET, reaching P<2x10-16 with 206,411 individuals.

Comment 2: *Selection of 11 mutations: Authors should rationalise why >= 30% penetrance and >= 2 OR was used to define high impact on obesity.*

Response 2: As suggested by the reviewer, we provide a more thorough justification for the chosen threshold. As such, we have added with a more detailed explanation to the **Supplemental Information** under the section “Penetrance and odds ratios to select high-impact mutations” (**P3 Par 4**). In addition, we briefly refer to these thresholds in the Results section (**P12 L20-25**).

Comment 3*: Top of Page 8: Physical activity according was not significant here (P = 0.08) in carriers. I think one aspect that has not been mentioned in the text is the impact of the other environmental risk factors which did seem to have different associations between carriers and non-carriers (smoking, physical activity).*

Response 3: The reviewer is correct that among carriers, the difference in physical activity between those of normal weight and those with obesity did not reach statistical significance, as reported at **P14 L23**. However, we believe the reviewer may be reading the results from Table 1 incorrectly, as the difference between carriers and non-carriers (last column) did not reach significance for either smoking (P=0.11 for normal weight, P=0.20 for obese) or physical activity (MET: P=0.60 for normal weight, P=0.91 for obese; IPAQ: P=0.20 for normal weight, P=0.45 for obese).

Reviewer 2:

Comment 1: *The most important limitation of this study is the use of genotype data to assess rare variants without confirmation of the variants. The authors report the use of the tool Evoker (Morris et al 2010) to ascertain the quality of the variants resulting in 59 variants of "good" quality. Evoker is being extended here from its original use for common variants. Based on the use of Evoker lite, Wright et al 2019 have noted that variants below MAF 0.001% are not reliably genotyped with the false positive rates ~60% in data from UKB, while those with MAF > 0.005% was ~20%. It is to be noted that 29 (out of 69) variants have MAF <= 0.001% including some from the 11 considered high penetrance and 59 variants have MAF <= 0.005%. Lotta et al 2019 used the same method for validation of the variants published in their paper, and the authors need to provide further evidence that these variants are true positives before perpetuating this method for ascertaining rare variants further.*
There is some evidence in the literature of the false-positive results of rare variants in Mendelian genes ascertained from genotyping data. In their own comparison of the subset of the sequencing

data with the corresponding genotype data, the authors postulate that 28 of the variants were deemed to be of low quality. While this reviewer acknowledges the limitation of scaling the results from analyses of ~10% of the data to the entire cohort. Most studies using exome data also validate the identified variants by one or other method prior to publication. Given the substantial implications of the conclusions of this manuscript, is it prudent to wait till the sequencing data for the entire UKB becomes available prior to making the assertion? If indeed a large number of variants are found to be false positive, would the conclusions still hold? The stochastic nature of such false positive findings makes it difficult to identify true positive amongst the variants noted in this study. It is possible that the conclusions of the study will hold after the due diligence of validation of the variants in which case, this publication will be applauded for accurate paradigm shifting conclusions. The influence of polygenic risk on BMI is not to be underestimated, especially when considering a similar effect in individuals who are not carriers of variants in MC4R. It is just not clear if the low polygenic risk is "protective" if the validity of the variants in MC4R is not established unequivocally.

Response 1: The reviewer raises an important concern, as the mutations' quality is critical to our analyses and conclusions. For that reason, we have implemented **rigorous and conservative quality control (QC) procedures** to ensure that the genotype data of the variants studied are of high quality and that our results are valid. Furthermore, we show in **sensitivity analyses** that, using the most stringent QC to retain variants in analyses, our conclusions will still hold.

As the reviewer points out, just using Evoker is likely not sufficient. The UK Biobank Access Team made the following recommendations, based on quality control procedures reported by others (Wright et al. 2019; Weedon et al 2019; Bycroft et al. 2018): "*In order to mitigate these inaccuracies, UK Biobank would suggest for rare variants, researchers should visually* ***inspect cluster plots****, both within and across batches, using an application such as Evoker*[4]*. For very rare variants, as the false positive rate is >80%, we recommend* ***checking the variants in individual-level data using the UK Biobank exome data***".

As such, we applied both recommendations for all our variants, rare and very rare, to ensure high-quality genotype data. We subsequently perform sensitivity analyses in which we remove variants of lower quality. These results are presented in **Table S2**, and described in detail in the **Supplemental Information**. Furthermore, we acknowledge the use of genotype-array data in the Discussion section (**P19 L1-9**). Specifically,

❖ **Inspection of cluster plots:** In brief, we generated cluster plots using Evoker for manual inspection by three investigators. We inspected the quality of the separation between heterozygous carriers and homozygous non-carriers and rated the clustering for each variant as "poor", "intermediate", or "good" as described in (Wright et al., 2019) and (Lotta et al., 2019). We have summarized the overall quality in **Table S2 (columns T-W) and in Supplemental Information (P4)**. As such, 10 variants were deemed of low quality (**Table S2, columns Y**) and were removed from downstream analyses. **All the 11 *MC4R* mutations with high penetrance and effect, key to the overall conclusion of our paper, were of high quality.**

❖ **Comparison of genotyped and sequenced data:** We examined the concordance between the genotyped and sequenced data in the subset of ~46,000 individuals for whom both data sources are available. The results of this comparison are shown in **Table S2 (columns J-S & X)** and described in **Supplemental Information (P4-5).** Thus, based on the comparison between genotyping and sequencing data in the subset of ~46,000 individuals, we identified at least 4 variants with an unacceptable False positive proportion (FPP >25%), another 22 variants for

which the FPP was calculated to be 100%, and 3 variants (one that overlapped with the 4 variants above) with an unacceptable false negative proportion (FNP >25%). Thus, the quality of the genotyped data for 28 variants was deemed to be low. We additionally considered the cluster plots for these 28 variants, because of the limitations of the FPP/FNP metric and because the sequenced subset consists of only ~10% of the full population analyzed. For 20 of these 28 variants, the cluster plots were of good quality. Therefore, we flagged these 20 variants, 3 of which are among the high impact mutations (**Table S2, column X**), and assessed their impact in a sensitivity analysis in which we remove them.

❖ **Sensitivity analyses:** Based on inspection of the cluster plots, all 11 variants were deemed of “good quality”, but two variants (rs1367004987, Affx-89021050) have high FPPs (>25%) and one variant (rs775382722) has a high FNP (>25%), based on the comparison between the genotyped and sequenced data in the 46K subset. Because this misclassification may have influenced our findings, we performed a sensitivity analysis in which excluded all three variants rs1367004987, Affx-89021050, and rs775382722. After excluding rs1367004987, Affx-89021050 and rs775382722, our sensitivity analysis – with 8 high-impact variants left – shows that the mean (SD) PRS values for carriers and non-carriers in normal weight and obese individuals continue to be robust (**Figure S1, Table S6**). Because the sample size of carriers is smaller and SE a little larger, P-values are a little higher ($P_{sens} = 1 \times 10^{-4}$ vs $P_{orig} = 1.7 \times 10^{-6}$). Difference for other characteristics remained very similar as well (**Table S6**). Furthermore, the additive effects of the 8 high-impact mutations and PRS on BMI are nearly the same (**Figures S1 & S2**).

Comment 2: *This manuscript focuses on individuals with overweight/obesity, while completely ignoring the principal of "people first language". People first language has been widely recognized as important for use in academic publications relevant to individuals with obesity (Kyle et al 2014, PMID 24616446, Wittert et al, PMID 26373880 and several others). The authors need to review their language throughout the manuscript, e.g. "normal weight carriers" will be carriers with normal weight, "obese carriers" will be carriers with obesity. "mutation carriers" should be carriers with mutations, "non-MC4R deficient individuals" should be individuals without mutations in MC4R etc. It is critical for scientists to remember that people are more important than pathology, always.*

Response 2: We agree with the reviewer and have made changes throughout the text.

Comment 3: *In the phenotype review, the authors have included the assessment of anthropometric parameters, but not the diagnoses codes or health status of the individuals. While the population enrolled in UK Biobank is expected to be healthier, for this manuscript, it will be important for the authors to review the ICD codes for the individuals under study to ensure that the "normal" weight was not due to an underlying illness? Further, as the authors are well aware that environmental influences far exceed indices of socioeconomic status and physical activity. How would the authors account for educational status as a measure of the socioeconomic awareness, smoking status and dietary habits in their modeling? Given the extensive phenotype data ascertained by UKB, it is naïve to include only age, sex and PCs derived from genetic data in the models. Additional phenotype data ought to be considered, at minimum for exploration or for sensitivity analysis.*

Response 3: We appreciate the reviewer’s insightful comment on ICD codes. As suggested, we have extracted the pre-enrollment ICD codes for the carriers of the 11 *MC4R* mutations who are of normal weight (N=28), but found that none had been assigned ICD codes that influence body weight with the exception of cancer diagnoses (4 participants), which may or may not have an impact on body weight.

The four participants had the following diagnoses:

	ICD10 code	Type of cancer	Time of occurrence
Individual 1	C61	malignant neoplasm of prostate	5 years prior to recruitment
Individual 2	C20	Malignant neoplasm of rectum	6 years prior to recruitment
Individual 3	C509	Malignant neoplasm of breast of unspecified site	3 years prior to recruitment
Individual 4	C445	Other and unspecified malignant neoplasm of skin of trunk	4 years prior to recruitment

Among the remaining 154 carriers, 11 individuals have had a ICD10 code for cancer diagnosis prior to recruitment. When comparing carriers of normal weight with all other carriers (P=0.26) or with carriers with obesity (P=0.24), we observed no significance differences in prevalence of these diagnoses.

We agree with the reviewer that the difference between carriers of normal weight and carriers with obesity may indeed be due to a number of non-genetic/environmental factors. The reviewer suggests to adjust our analyses for these environmental factors. As our aim was to assess the contribution of (poly)genetic, as well as non-genetic/environmental factors, to the difference between carriers of normal weight and those with obesity, we chose not to adjust for these factors in our primary analyses. We report these findings in the Results section (**P14 L12-15**) and in **Table 1**. However, as the reviewer suggested, we have now performed a secondary analysis in which we compare the PRS_{BMI} between carriers of normal weight and carriers with obesity, adjusting for physical activity, smoking, and the townsend deprivation index (socio-economic status), in addition to age, sex, and the first 10 principal components. We present these findings in **Table S7**, showing that the difference in PRS_{BMI} observed between carriers of normal weight and carriers with obesity is reduced by ~10% after additionally adjusting for physical activity, smoking, and the townsend deprivation index, from ~1.04 SD to 0.94 SD. These findings support the notion that part of the difference between carriers of normal weight and those with obesity is due to non-genetic/environmental factors, whereas part is due to genetic factors. We describe this secondary analysis in the Methods (**P11 L17-18**) and Results (**P14 L16-17**) sections.

Comment 4: *Page 6: "For 10 (91%) of the 11 mutations, there was evidence that the mutation impaired MC4R function and/or led to reduced activity, based on functional characterization (Tables S3 and S4), which was significantly more often than for the remaining 48 mutations (P = 0.0006) for which we found evidence for only 17 (33%)." In table S3, there are 20 variants that have been reported to have functional effect while the text mentions 17. Which one is correct?.*

Response 4: We thank the reviewer for noticing this inconsistency, which we had failed to update before submission. The results in **Table S3** were/are correct and inconsistencies between tables and text (**P13 L6-7**) have now been resolved.

Comment 5: *Authors need to discuss the limitations of using recall data from 10 years of age for adults recruited at 40-60 years of age, especially with reference to a phenotype such as height and weight and be conservative in their conclusions from such data in the abstract.*

Response 5: As suggested, we acknowledge the use of self-reported recall data in the Methods (**P8 L11-15**), Results (**P14 4-7**), and Discussion section (**P17 L12-20**) and have adapted the language related to conclusions based on these observations.

Comment 6: *Page 7: "These observations suggest that normal weight carriers are able to overcome their increased obesity risk due to MC4R mutations, at least in part, thanks to a low polygenic risk." Scientific articles should not be using language that glorifies one weight category over the other. There are a few other instances where colloquial language is used: "the extent to which people's polygenic risk (PRSBMI) affects BMI and obesity risk among carriers and non-carriers"*

Response 6: As suggested, we have modified this sentence (**P14 L19-20**) and revised others to avoid glorification of one weight category over another.

Reviewer 3:

Comment 1: *This study is not easy to read for clinicians and biologist unless they have a strong background in modern genetics and statistics. My recommendation is to provide both graphics and explanations to better guide the reader throughout the experimental design. A scheme putting into evidence the main questions addressed as well as the relative answers should be included.*

Response 1: We appreciate the reviewer's suggestion and have summarized our study design in a flowchart that we will include as a **Figure 1**. We refer to the flowchart in the Methods section at **P10 L17**.

Comment 2: *The 11 mutations with high impact are selected based on penetrance (>30%) and obesity risk (increase>2 fold). More in deep and detailed explanations should be provided to explain these criteria which may sound arbitrary otherwise.*

Response 2: As suggested by the reviewer, we provide a more thorough justification for the chosen threshold. As such, we have added with a more detailed explanation to the **Supplemental Information** under the section "Penetrance and odds ratios to select high-impact mutations" (**P3 Par 4**). In addition, we briefly refer to these thresholds in the Results section (**P12 L20-25**).

Comment 3: *The last column of table 1 as well as of the supplementary tables indicate the P value when carriers and non carriers are compared. The sample size of these groups differ by various order of magnitude. Is it right to compare groups with so different sample size , i.e 29 versus > 100000? Provide explanations*

Response 3: The reviewer is correct that sample sizes between carriers and non-carriers are unbalanced. Therefore, we used the Welch's t-test to compare the means for continuous traits and the Fisher's exact test to compare of categorical/ordinal data. Both tests are used when sample sizes are unequal. We have clarified this in the Methods section (**P11 L19**).

Reviewer 4:

Comment 1: *The authors do acknowledge that UK Biobank represents a healthier slice of society. Given that deprivation is inversely correlated with health, how does the deprivation index of UK biobank compare to other large population cohort studies?*

Response 1: The reviewer is correct that also deprivation affect populations' health. In a recent study, Weng et al (PLoS ONE 2019) showed that UK Biobank population was significantly less deprived, based on the Townsend deprivation index, compared to the 2001 UK census data. We have included this information in the Discussion section (**P18 L26**) and added a relevant citation.

Comment 2: *While the carriers with a lower polygenic risk score have their MC4R genetic risk*

mitigated, does the specific SNP (rs571312) near MC4R play any increase or decreased role in this mitigation?

Response 2: The C-allele (allele frequency ~76%) of rs571312 has been associated with a lower risk of extreme and early onset obesity, by Wheeler et al (Nature Genetics 2013). We found that 19 of 28 MC4R mutations carriers of normal weight (69%) carry this protective allele, compared to 45 of 76 MC4R carriers with obesity (59%). While the protective allele is indeed somewhat more frequent among the normal weight carriers than among obese carriers, this difference was not significant (P=0.4). Furthermore, the difference in PRS_{BMI} between carriers of normal weight and carriers with obesity remained unchanged after additionally adjusting for rs571312. Therefore, we do not believe this variant plays a role in the mitigation seen in our observations.

Comment 3: *I apologise if this is an ignorant question, but in table 1, what exactly are adjusted standardized scores?*

Response 3: We use standardized scores, which allow for direct comparisons of the effect sizes across traits, as they are all on the same scale. The “raw” values on the original scale are shown in Supplementary Table S1. We explain how these values should be interpreted in the legend of **Table 1**.
