# Supplementary material for: The role of polygenic susceptibility to obesity among carriers of pathogenic mutations in MC4R in the UK Biobank population
Source: PLoS Med. 2020 Jul 21;17(7):e1003196. doi: 10.1371/journal.pmed.1003196 (PMC7373259; doi:10.1371/journal.pmed.1003196)
Supplement: Attachment — Submitted filename: PMEDICINE-D-20-00082R3 - RESPONSE TO PRODUCTION TEAM - FINAL.pdf [file pmed.1003196.s014.pdf]

REQUESTS FROM THE PRODUCTION TEAM:

Comment: *1. Tables 2 and 3 use returns to control text wrapping. This is indicated by a [¶ or ↲] symbol when "Show Paragraph Marks" is turned on in Word. Please either split those cells into separate cells to achieve alignment or remove the returns.*

Response: I have removed the returns in Tables 1 and 2 (which we assumed you meant).

Comment: *2. Please ensure that the funders and grant numbers match between the Financial Disclosure field and the Funding Information tab in your submission form. Note that the funders must be provided in the same order in both places as well.*

Response: Both fields are now consistent.

Comment: *3. Please ensure that all Supporting Information files use the following format verbatim throughout the manuscript (including legends and all in-text citations): S1 Fig, S1 Table, S1 Text, S1 Data, etc. Each file type should start at 1 and increase numerically. Please note, however, that the file names themselves must use an underscore rather than a space, i.e. S1_Fig.tif, S1_Table.xlsx, S1_Text.doc, S1_Data.xlsx, S1_PRISMA_Checklist.docx, etc.*

Response: We have double-checked the labelling and believe all are consistent with the Journal's requirements.

Comment: *4. Thank you for addressing the requirements of the journal. However, we note that much of the stigmatising labels remain in Table 1 and throughout the manuscript.*
We appreciate the need to accurately represent the questionnaire data, however please note that questionnaires are designed and validated for users and not necessarily designed to be used verbatim in scientific articles. In this case, it appears that the UK biobank questionnaires do not adhere to people first language principles but as a journal with a global readership we need to adhere to these principles.
To this end, I ask that you change the language as requested in the previous decision letter in order to proceed with your publication. In addition, please provide the questionnaires referred to in the methods as references 18-21 do not contain the questionnaires mentioned.

Response: We have changed the labels related to the questionnaire in the text and in Table 1, as well as in supplementary tables. We have double-checked the labelling to avoid stigmatizing and updated text and supplementary information according.

We have also include a website reference that takes readers to the UK Biobank questionnaires.
